# Endotoxin content in neonatal formulas, fortification, and lactoferrin products: association with outcomes and guidance on acceptable limits

**DOI:** 10.1007/s10534-022-00487-1

**Published:** 2023-01-27

**Authors:** David A. Kaufman, Patti H. Perks, Rachel G. Greenberg, David Jensen

**Affiliations:** 1 University of Virginia, Charlottesville, VA USA; 2 Duke Clinical Research Institute, Durham, NC USA; 3 Duke Office of Regulatory Affairs and Quality, Durham, NC USA

**Keywords:** Endotoxin, Lipopolysaccharide, Preterm infants, Lactoferrin, Formula, Fortifiers

## Abstract

While endotoxin (lipopolysaccharide) can be harmful and contribute to morbidity and mortality with Gram-negative sepsis or necrotizing enterocolitis in preterm infants, non-toxic amounts are produced as part of the neonatal microbiome and may be present in enteral nutrition and medications administered. The United States Food and Drug Administration has given guidance for endotoxin concentration limits for intravenous medications and fluids of 5 endotoxin units/kg/hour (120 endotoxin units/kg/day), but no guidance for amounts of endotoxin in enteral products. To determine baseline exposure to infants in the neonatal intensive care unit, we examined endotoxin content of enteral formulas and fortification used for preterm infants, as well as bovine lactoferrin products. We also examined endotoxin exposure and outcomes in very low birth weight infants. Endotoxin content was measured using kinetic chromogenic limulus amebocyte lysate analysis. Daily endotoxin exposure from enteral formulas ranged between < 75 to 7110 endotoxin units/kg and from lactoferrin products from 7 to 3720 endotoxin units/kg. In examining neonatal outcomes from a bovine lactoferrin product studied at three different escalating doses (100, 200, and 300 mg/kg/day), we measured endotoxin in the lactoferrin product and daily exposure was 1089 (*N* = 10), 2178 (*N* = 10) and 3287 (*N* = 11) endotoxin units/kg, respectively. There were no cases of necrotizing enterocolitis or mortality and no lactoferrin-related adverse effects in these patients. Enteral endotoxin daily exposures from lactoferrin products are similar to amounts in preterm enteral nutrition and appear safe and not associated with patient harm. Testing enteral products and establishing safety limits may improve care of high risk patients.

**Supplementary Information:**

The online version contains supplementary material available at 10.1007/s10534-022-00487-1.

## Introduction

While enteral endotoxin or lipopolysaccharide (LPS) can be associated with bowel injury that may lead to microbial gut translocation and necrotizing enterocolitis (NEC), low levels may be non-toxic and are present from the microbiome and in enteral products administered to neonates. (Hsueh et al. 2003; Wu et al. 2021) The United States Food and Drug Administration (FDA) has given guidance for an intravenous endotoxin dose threshold of 5 EU (endotoxin unit)/kg/hour which would equate 120 EU/kg/day, but there is no guidance for enteral intake of endotoxin for enteral nutrition or medications. (FDA 2014**; **USP 2012**)** Furthermore, enteral nutrition products including preterm formulas, do not routinely report endotoxin as part of their manufacturing and release criteria. (Wassenaar & Zimmermann 2018) (Wakabayashi et al. 2018) While products undergo pasteurization to kill pathogens, endotoxin/LPS is heat stable and remains intact and present in the product after pasteurization. (Allen 2022; FDA 2014; McCollough & Radhakrishna Tirumalai 2018).

A few studies have examined endotoxin content in milk products and infant formulas. Townsend et al. examined endotoxin content in 31 powdered infant formulas and found 40 to 55,000 EU per gram with an estimated daily exposure ranging from 67 to 97,670 EU/kg. (Townsend et al. 2007; Wu et al. 2021) Recently, another study found endotoxin content in infant formulas ranged between 6 and 9080 EU/ml for 149 reconstituted samples with an estimated daily exposure (based on 150 ml/kg/d enteral intake) ranging from 900 to 1,362,000 EU/kg. (Wu et al. 2022) Comparatively, animal models of NEC that utilize endotoxin require a minimum of 1,000,000 EU/kg in addition to other stressors such as hypoxia and hypothermia.(Ren et al. 2019) (Hsueh et al. 2003; Zhang et al. 2020) While a healthy infant gut, mature immune system and intestinal barrier can contain certain amounts of endotoxin, preterm and sick newborns or its with gut disease may not be able to contain the same amounts. (Wu et al. 2021) While these studies established that endotoxin is present and the varying amounts that are contained in infant formulas, they did not examine preterm infant formulas where certain endotoxin amounts potentially could cause harm compared to formulas given to older infants during the first year after birth.

To establish a baseline of how much endotoxin preterm infants may be receiving and tolerating, we examined the endotoxin content in enteral nutrition used for preterm infants and infants with gastrointestinal disease, feeding intolerance, malabsorption and dysmotility. We also examined the amount of endotoxin in several lactoferrin products. Additionally, the effect of endotoxin exposure on neonatal outcomes was evaluated from one bovine lactoferrin study in preterm infants where the lactoferrin endotoxin content was measured to provide a risk assessment of the potential immunomodulatory effect.

## Materials and methods

We examined 11 enteral formulas and two bovine-based human milk fortifiers (HMF). Eight were liquid formulations: Similac® Special Care® Premature 24 (whey concentrate), Similac® Special Care® Premature 30 (whey concentrate), Enfamil® Premature 24 (whey concentrate), Similac® Neosure® 22 (whey concentrate discharge formula), Similac® for Spit up (milk protein isolate), Pregestimil® 20 (extensively hydrolyzed casein), Nutramigen® (extensively hydrolyzed casein), Alimentum® (extensively hydrolyzed casein) and three were powdered formulations: Similac® Neosure® 22 (whey, discharge formula), Nutramigen® Enflora™ with LGG®(extensively hydrolyzed casein), Elecare® (amino acid). The two HMFs evaluated were Similac® HMF (casein hydrolysate) and Enfamil® HMF (whey hydrolysate).

Ten bovine lactoferrin products from five manufacturers were analyzed for endotoxin: whey derived products from Glanbia nutritionals (Bioferrin®, USA) and skim milk derived products from Tatua (New Zealand), Hilmar (California, USA), FrieslandCampina (Vivinal®, The Netherlands) and Dicofarm (BLF 100, Italy).

Endotoxin content was measured for each sample (lot) in duplicate using kinetic chromogenic LAL (limulus amebocyte lysate) analysis performed by Charles River Laboratories, Charleston, SC. Dilution in 50% Endotoxin Specific Buffer and 2% saline with heat treatment at 75 °C for 10 min was performed to eliminate any potential interfering substances. Analysis was performed in March 2020 and July 2021.

Neonatal outcomes were examined from a bovine lactoferrin study in very low birth weight infants in which we measured lactoferrin endotoxin content and correlated endotoxin exposure with outcomes. In that study, bovine lactoferrin (Glanbia Nutritionals, USA) was administered enterally at escalating doses of 100, 200, or 300 mg/kg per day to three study groups providing three different endotoxin exposure amounts. Outcomes, except for mortality, were evaluated during the 30 day treatment period through 7 days after the last dose. Mortality was examined during the entire hospitalization. Additional study details can be found in the trial publications (Itell et al. 2020; Kaufman et al. 2020).

Endotoxin content in formulas and fortifiers was reported at EU/ml. These were either liquid or powder products prepared with endotoxin-free water. For daily endotoxin contents, enteral intakes of 150 ml/kg were used to calculate amounts received over a 24 h period. Endotoxin for lactoferrin products was reported as EU/mg and the daily endotoxin amount in lactoferrin was based on 300 mg/kg dosing of lactoferrin since it is the amount received in colostrum per 24 h. (Mastromarino et al. 2014; Sherman et al. 2012; Villavicencio et al. 2017).

## Results and discussion

Endotoxin was detected in most formulas (Table 1). There was a variation in endotoxin content of enteral formulas used in the NICU with a mean of 7.3 EU/ml and potential infant exposure averaging 138 EU/kg/feed (if feeding every 3 h) and 1102 EU/kg over a 24 h period (Table 1). Preterm infant formulas had a mean of 7.9 EU/ml that would contain 148 EU/kg/feed (if feeding every 3 h) and 1184 EU/kg/24 h. There was no statistical difference between lots, brands or date of testing. Supplementary Table 1 includes the results of each formula lot evaluated.

**Table 1:** Endotoxin content in enteral nutrition used in the neonatal intensive care unit

	Endotoxin content (EU/ml)	Daily exposure over 24 h (EU per kg)
All formulas (*N* = 22)	7.3 (< 0.5–47.4)	1102 (< 75–7110)
Preterm enteral formulas (*N* = 7)	7.9 (0.97–25.8)	1184 (145–1695)
Extensively hydrolyzed casein-based formulas (*N* = 8)	1.3 (< 0.5–5.6)	193 (< 75–840)
Amino acid based formula (*N* = 1)	1.21	182
Discharge Formulas (*N* = 5)	18.8 (3.3–47.4)	2824 (495–7110)
Human milk fortifiers (*N* = 3)	1.9 (< 0.5–4.4)	61 (< 25–110)

Mean (range)

*N* = number of lots tested

“ < ” indicates lower limit of detection

Formula EU per day based on 150 ml/kg/day enteral intake

Fortifier EU per day based on HMF supplementation for 150 ml/kg/day enteral intake of human milk

Endotoxin mean content in lactoferrin products was 2.55 EU/mg and daily exposure ranged from 7 to 3720 EU/kg/day based on lactoferrin dosing of 300 mg/kg/day with higher levels in whey derived products compared to skim milk derived products (Table 2).

**Table 2:** Endotoxin content of lactoferrin (LF) products and estimated daily intake based on dosing of 300 mg/kg per day

Lactoferrin products	EU/mg	Daily EU/mg per kg exposure over 24 h based on 300 mg/kg/day intake of LF
All	2.55 (0.022–12.4)	765 (7–3720)
Skim milk derived LF products	0.27 (0.022–0.888)	81.5 (7–266)
Whey derived LF products	11.7 (10.9–12.4)	3496 (3270–3720)
FrieslandCammpina (lot A)	0.022	7
FrieslandCammpina (lot B)	0.059	18
Tatua (lot A)	< 0.05	< 15
Hilmar (lot A)	0.14	42
Hilmar (lot B)	0.174	52
Hilmar (lot C)	0.339	102
Hilmar (lot D)	0.888	266
Dicofarm (lot A)	< 0.500	< 150
Glanbia (lot A)	10.9	3270
Glanbia (lot B)	12.4	3720

Mean (range), “<” indicates lower limit of detection

In examining the endotoxin content (10.9 EU/kg) of the bovine product we studied in very low birth weight infants, the highest endotoxin exposure was 3720 EU/kg/day which was similar to formulas used for preterm infants. There were no cases of necrotizing enterocolitis (NEC), serious adverse effects, or mortality during hospitalization in 31 patients (Table 3). While no other neonatal lactoferrin study examined endotoxin content, there was no increase in NEC, infections, mortality or other lactoferrin-related adverse effects reported in the two largest studies or systematic reviews. (Griffiths et al. 2018; Pammi & Suresh 2020; Tarnow-Mordi et al. 2020).

**Table 3:** Endotoxin exposure with 3 different lactoferrin doses and clinical outcomes in very low birth weight infants (< 1500 g)

Bovine LF Groups	100 mg/kg (*N* = 10)	200 mg/kg (*N* = 10)	300 mg/kg (*N* = 11)
Endotoxin exposure (EU/kg/day)	1089	2178	3267
Gestational age (weeks) [Mean (range)]	29.4 (24.1–34.0)	29.1 (24.6–32.7)	26.6 (24.3–30.9)
Birth weight (grams) [Mean (range)]	1130 (590–1450)	1350 (850–1480)	900 (540–1480)
NEC (≥ stage 2)	0	0	0
Focal bowel perforation	0	0	0
Bloodstream infection	1	1	1
Urinary tract infection	0	0	0
No. of sepsis evaluations	4	4	4
Full enteral feeds reached (day after birth) [Mean (range)]	13 (8–19)	13 (7–28)	13 (10–29)
No. of episodes enteral feedings were held for > 24 h	2	2	2
Lactoferrin- related SAEs	0	0	0
Mortality during entire hospitalization	0	0	0

The amounts of endotoxin present in formulas and lactoferrin are low and from our data is not associated with gut injury or NEC in very low birth weight infants. In animal models of NEC using endotoxin/LPS, a significantly higher amount is required to cause gut wall injury and NEC and often with additional stressors of hypoxia and hypothermia (Choi et al. 2010; Hsueh et al. 2003). LPS is measured by endotoxin units. One endotoxin unit (EU) equals approximately 0.1 to 0.2 ng of endotoxin per ml and represents the amount of LPS produced by ~ 100 *E. coli* bacteria. Therefore, 1 mg of endotoxin is equal to 5,000,000 to 10,000,000 EUs. In rodent, rabbit and porcine animal models, a minimum of 1 mg or 10,000,000 EU/kg is needed to produce NEC diagnosed by pathologic examination(Hsueh et al. 2003; Ren et al. 2019; Zhang et al. 2020). Study of lower doses of endotoxin of 5,000,000 EU/kg did not cause NEC in these animal models (Hsueh et al. 2003). It is important to note that the human gut may respond differently than these informative animal models.

Comparatively, the endotoxin amounts in preterm formulas and lactoferrin per 24 h were significantly lower compared to animal models causing NEC. Depending on the formula or lactoferrin product, the endotoxin content is approximately 10,000- to 1,000,000-fold lower than amounts needed to cause gut injury or NEC.

There is some evidence that low amounts of endotoxin may be beneficial for both the host and its microbiome (Wu et al. 2021). Animal studies have shown that low endotoxin amounts may have a probiotic effect, reduce allergic diseases, and aid immune development (Inagawa et al. 2011; Smit et al. 2009; Taniguchi et al. 2009; Wassenaar & Zimmermann 2018). Further study of this area is needed as safe amounts of endotoxin may depend on gut health and host immunity.

Determining “physiologic” endotoxin content in the neonatal gastrointestinal tract is complex and influenced by many factors. Some important neonatal factors include gut microbiome diversity, endothelial tight junction health, and the presence of other molecules, such as lactoferrin, which binds endotoxin (Sochaczewska et al. 2022; Van Saene et al. 1992). Future studies should examine endotoxin stool levels with gut function and diseases as well as neonatal infectious related outcomes in preterm infants.

Endotoxin/LPS also has a complex interaction with Toll-like receptor-4 (TLR4). The TLR4 gene is programmed to not react with LPS derived from commensal bacteria, so intestinal epithelia inflammation does not occur in this environment (Takahashi et al. 2009). In contrast, when the intestinal barrier is compromised or leaky, LPS may be able to translocate past the intestinal barrier and interact with immune cells leading to inflammation.

As discussed above, there is no guidance on enteral endotoxin limits as there are for intravenous products. Establishing enteral thresholds would improve the safety of milk and milk-derived products used in the NICU and for any patient at high risk for harm from endotoxin. Several factors in the collection, management and production of milk and milk-derived products can affect endotoxin content (Wu et al. 2021). While pasteurization eliminates bacteria, if there are large amounts of Gram-negative bacteria present at the time of pasteurization, high amounts of endotoxin will be released into the milk. After milk collection, milk can be stored in milk tanks and then transported at temperatures in which some Gram-negative bacteria (e.g. *Pseudomonas* and *Acinetobacter*) can proliferate yielding higher amounts of endotoxin being released when milk is sterilized. Gram-negative bacteria can also proliferate when infant milk powder is brewed. Additionally, fermentation as part of formula manufacturing also can affect bacterial content (Granier et al. 2013; Wallace et al. 2016). Microbial content of milk can be decreased by reducing microbial pollution of pastures, shorten or eliminate time milk is in milk tanks and transportation time, and reduce heat injury of important components such as alkaline phosphatase, which helps decompose endotoxin, as well as lactoferrin, which binds endotoxin.

Limitations of this analysis is that since infants were receiving lactoferrin, which binds endotoxin, it may have neutralized side effects of endotoxin. Furthermore, endotoxin detected in the lactoferrin products may have represented endotoxin bound to lactoferrin that was released during testing versus “free endotoxin” that may interact with the gut mucosa.

In conclusion, daily amounts of enteral endotoxin in formulas and lactoferrin products we tested are low compared to amounts that cause gut injury and NEC in animals. Furthermore, these endotoxin amounts when given with lactoferrin were not associated with gut injury, dysmotility, NEC or other adverse effects. Since there is no guidance for enteral intake of endotoxin for enteral nutrition or medications**,** this information and future studies may be able to aid in the establishment of recommendations**.** Knowledge of endotoxin content of enteral nutrition and medications used in the NICU may help make care of high risk infants safer.

## Supplementary Information

Below is the link to the electronic supplementary material.

Supplementary file1 (DOCX 15 KB)
