# Supplementary material for: Endotoxin content in neonatal formulas, fortification, and lactoferrin products: association with outcomes and guidance on acceptable limits
Source: Biometals. 2023 Jan 27;36(3):703–8. doi: 10.1007/s10534-022-00487-1 (PMC10181959; doi:10.1007/s10534-022-00487-1)
Supplement: Supplementary file 1 — Supplementary file1 (DOCX 15 KB) [file 10534_2022_487_MOESM1_ESM.docx]

**Supplementary Table**

| **Formula or Fortifier** | **Endotoxin Units (EU)/ml** | **EU/kg per**  **24 hours** |
| --- | --- | --- |
| **March 2020 testing of each lot** |  |  |
| Pregestimil® 20  (casein) | <0.500 | 0 |
| Nutramigen® Enflora™ with LGG®  (casein) | <0.500 | 0 |
| Nutramigen®  (casein) | 1.2 | 144 |
| Similac® Neosure® 22  (milk, whey) | 3.3 | 396 |
| Enfamil® Premature 24  (milk, whey) | 4.5 | 540 |
| Alimentum®  (casein) | 5.6 | 672 |
| Similac® Special Care® Premature 24 (milk, whey) | 9.7 | 1164 |
| Similac® Special Care® Premature 30 (milk, whey) | 11.3 | 1356 |
| Similac® Neosure® 22  (milk, whey) | 21.4 | 2568 |
| Similac® Special Care® Premature 24 (milk, whey) | 25.8 | 3096 |
| Similac® Neosure® 22  (milk, whey) | 47.4 | 5688 |
| **Bovine Human Milk (HMF) Fortifiers** |  |  |
| Similac® HMF (casein hydrolysate) | <0.500 | 0 |
| Enfamil® HMF (whey hydrolysate) | 4.40 | 110 |

| **Formula or Fortifier** | **Endotoxin Units (EU)/ml** | **EU/kg per**  **24 hours** |
| --- | --- | --- |
| **July 2021 testing of each lot** |  |  |
| Similac® Special Care® Premature 24 (milk, whey) | 0.969 | 145 |
| Similac® Special Care® Premature 24 (milk, whey) | 1.47 | 221 |
| Similac® Special Care® Premature 30 (milk, whey) | 1.49 | 224 |
| Similac® Neosure® 22 (milk, whey) | 15.1 | 2265 |
| Similac® for Spit up (milk protein isolate) | 0.804 | 121 |
| Alimentum® (extensively hydrolyzed casein) | <0.500 | <75 |
| Nutramigen® (extensively hydrolyzed casein) | <0.500 | <75 |
| Pregestimil® 20 (extensively hydrolyzed casein) | <0.500 | <75 |
| Nutramigen® Enflora™ with LGG® (extensively hydrolyzed casein) | <1.00 | <150 |
| Similac® Neosure® 22 (milk, whey) | 6.93 | 1040 |
| Elecare® (amino acid) | 1.21 | 182 |
